# Preparedness for Life-Threatening Situations in a Pediatric Tertiary-Care University Children’s Hospital: A Survey †

**DOI:** 10.3390/children9020271

**Published:** 2022-02-16

**Authors:** Francis Ulmer, Sabine Pallivathukal, Andreas Bartenstein, Ruth Bieri, Daniela Studer, Sebastiano A. G. Lava

**Affiliations:** 1 Paediatric Intensive Care Unit, Department of Paediatrics, University Children Hospital of Berne, Inselspital, and University of Bern, 3010 Bern, Switzerland; francis.ulmer@insel.ch (F.U.); ruth.bieri@insel.ch (R.B.); daniela.studer@insel.ch (D.S.); 2 Paediatric Cardiology Unit, Center for Congenital Heart Disease, Department of Cardiology, Inselspital, Bern University Hospital, and University of Bern, 3010 Bern, Switzerland; sabine.pallivathukal@insel.ch; 3 Department of Paediatric Surgery, University Children Hospital of Bern, Inselspital, and University of Bern, 3010 Bern, Switzerland; andreas.bartenstein@insel.ch; 4 Paediatric Cardiology Unit, Department of Paediatrics, Lausanne University Hospital and University of Lausanne, 1011 Lausanne, Switzerland; 5 Division of Clinical Pharmacology and Toxicology, Institute of Pharmacological Sciences of Southern Switzerland, Ente Ospedaliero Cantonale, 6900 Lugano, Switzerland; 6 Heart Failure and Transplantation, Department of Paediatric Cardiology, Great Ormond Street Hospital, London WC1N 3JH, UK

**Keywords:** cardiopulmonary resuscitation, preparedness, training needs, shock, arrhythmias, airway, emergency, life-threatening situation

## Abstract

Pediatric nurses and physicians are rarely exposed to life-threatening events. Understanding the needs of clinicians is key for designing continuing training programs. A survey exploring preparedness to manage life-threatening events as well as training needs was mailed to all clinically active nurses and physicians at a tertiary-level referral children’s hospital. Overall, 469 participants out of 871 answered the questionnaire (54% response rate). Respondents felt well or very well (nurses 93%, physicians 74%) prepared to recognize a deteriorating child and rated their theoretical understanding (70% well or very well prepared) of how to manage life-threatening situations significantly higher (*p* < 0.0001) than their cardiopulmonary resuscitation (CPR) preparedness (52% well or very well prepared). Both perceived theoretical understanding (*p* < 0.0001) and CPR preparedness (*p* < 0.002) were rated higher among nurses than physicians. Arrhythmias, shock, cardiac arrest and airway management constitute main areas of perceived training need. In conclusion, although a majority of pediatric nurses and physicians felt sufficiently trained to recognize a deteriorating child, their perceived ability to actively manage life-threatening events was inferior to their theoretical understanding of how to resuscitate a child. A high degree of institutional confidence and identification of areas of training need provide a good foundation for customizing future continuing education programs.

## 1. Introduction

Pediatric healthcare providers’ routine in dealing with acute care situations is limited to infrequent exposures, as life-threatening events are rare in pediatrics [1,2,3]. However, pediatric hospitalists need to be trained to competently face these emergencies [3,4]. Similarly, recurring training in acute care management is recommended as part of primary and continuing nursing education [5]. Emergency preparedness among pediatric healthcare professionals has sadly been described as unsatisfactory [6]. 

In an effort to explore self-perceived acute care readiness and training needs among nurses and physicians, a survey among clinically active staff of a tertiary-level university children’s hospital was performed. 

## 2. Materials and Methods

E-mail invitations containing the questionnaire were sent to all clinically active nurses and physicians working in the departments of pediatrics, including Pediatric Emergency Medicine, Pediatric Intensive Care, Neonatology, and Pediatric Surgery of the University Children’s Hospital of Berne, Switzerland. This tertiary-level referral children’s hospital cares for approximately 22,000 outpatients, 18,000 emergency consultations and 4000 inpatients (including approximately 700 newborns and 700 intensive care admissions) per year. 

Participation was voluntary and anonymous; the completion of the survey was deemed as consent to participate. The questionnaires could be printed, filled in and returned in anonymous boxes placed inside the hospital. The survey instrument (Supplementary Material Table S1) elicited information on the following issues: (1) demographic and baseline information (professional experience, employment duration at the hospital, weekly working hours, frequency of life-threatening patient encounters); (2) self-perceived readiness to face life-threatening situations and preparedness in operating medical devices relevant to their occupational environment; (3) perceived quality of care; and (4) perceived training needs pertaining to specified clinical settings, applicable to simulation training (management of arrhythmias, emergency medications, head trauma, cardiac arrest, cardiopulmonary resuscitation, mask ventilation, meningitis, multisystem trauma, seizures, severe dehydration, shock and airway management).

The 10-item, cross-sectional survey was designed over a series of developing conferences among authors, according to survey research recommendations [7,8,9,10,11] and following a pre-defined procedure [12,13]. Questions were selected according to pertinence and clinical relevance, as well as the possibility of answering them in a close-ended, unambiguous way. Since previous research is limited and not transferrable to our setting, a formal validation procedure was not possible. However, the initial version of the close-ended questionnaire was iteratively refined, piloted among eight physicians and seven nurses and edited in accordance with the obtained feedback. The pilot also tested if the questionnaire could be completed within 10 min. The survey was independent and not commercially sponsored. Participants could not be retraced.

Ordered categorical responses were assigned a numerical score [14,15] and analyzed using a nonparametric analysis of variance (Mann–Whitney U-test for independent samples). Proportions were analyzed with Fisher’s exact test. Significance was assigned at *p* < 0.05 (two-tailed).

## 3. Results

The questionnaire was answered by 469 (54%) out of 871 invited health professionals. The response rate was significantly higher among nurses (69%) than physicians (37%, *p* < 0.0001). Each question was answered by at least 453 participants (97%). The baseline characteristics of respondents are depicted in Table 1. Nurses reported significantly more work experience (*p* = 0.001), whereas physicians reported longer working hours (*p* < 0.0001) and more frequent encounters with life-threatening situations (*p* = 0.0024).

**Figure 1:**
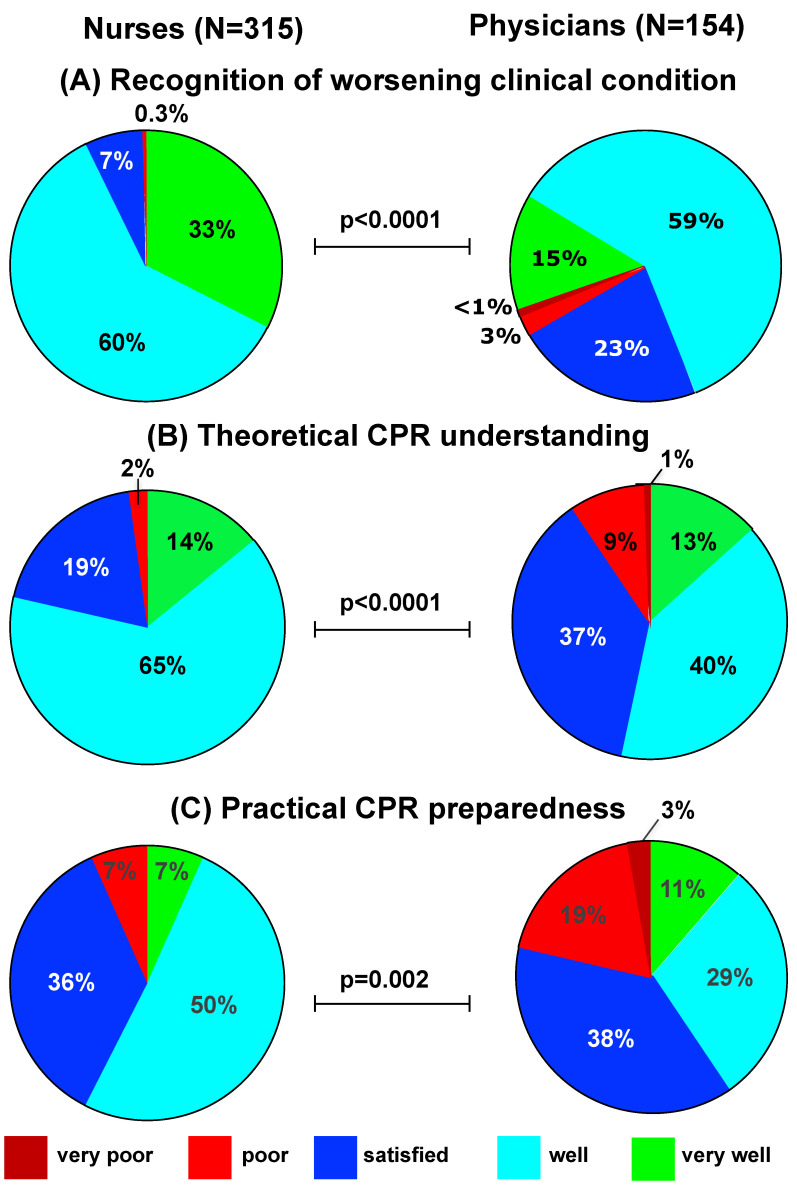
Self-perceived readiness to recognize a clinical deterioration and perform cardiopulmonary resuscitation among nurses and physicians. Nurses, significantly more often than physicians, felt well or very well prepared to recognize a worsening clinical condition of a child (**A**). Similarly, nurses rated their theoretical understanding (**B**) and practical (**C**) preparedness to manage a life-threatening situation requiring cardiopulmonary resuscitation (CPR) significantly higher compared to physicians.

Overall, 74% of physicians and 93% of nurses (*p* < 0.0001) considered themselves to be well or very well prepared to recognize worsening clinical conditions (Figure 1A). 

**Figure 2:**
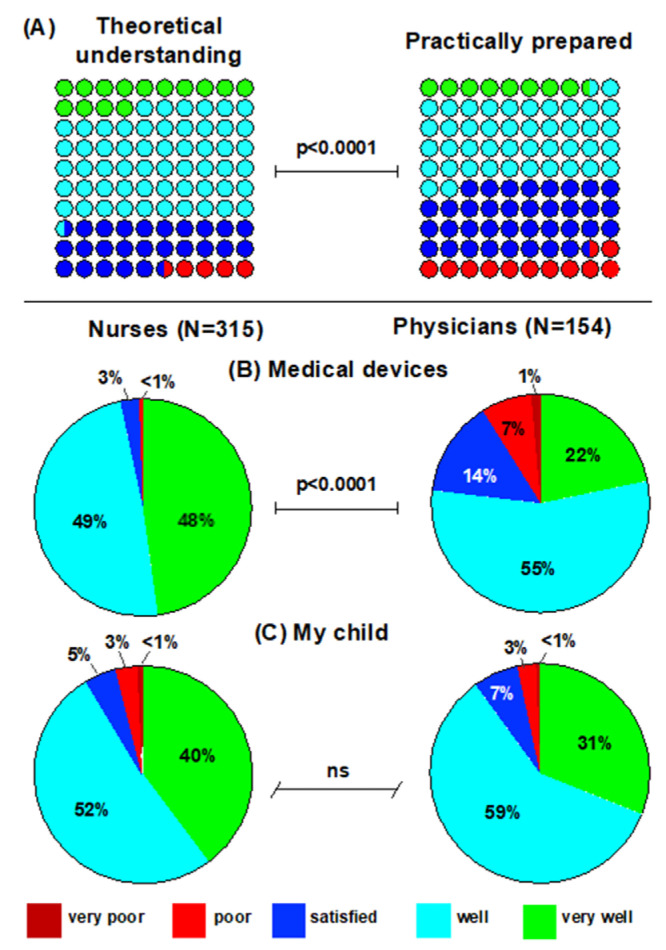
Self-perceived theoretical and practical competence, skills in using medical devices and confidence in the institutional quality of acute care. Participants from both professional groups were much more likely to state they felt well or very well prepared to manage life-threatening situations in theory than in practice (**A**). Nurses more frequently than physicians felt well or very well trained to operate medical devices in their work environment (**B**). Both professional groups felt safe to have their own child cared for at the hospital in which they were working (**C**).

In total, 79% of nurses and 53% of physicians believed they had a good or very good theoretical understanding of how to approach acute care situations necessitating cardiopulmonary resuscitation (*p* < 0.0001, Figure 1B). Similarly, 57% of nurses but only 40% of physicians felt they were well or very well prepared to manage an acute care situation requiring them to perform cardiopulmonary resuscitation (*p* = 0.002, Figure 1C). Overall, participants felt better prepared in theory than in practice (*p* < 0.0001, Figure 2A). This difference was significant among both nurses (*p* < 0.0001) and physicians (*p* = 0.0089).

Overall, 97% of nurses but only 77% of physicians stated they felt well or very well trained to operate medical devices in their work environment (*p* < 0.0001, Figure 2B). Interestingly and reassuringly, 91% of participants stated they would feel safe or very safe to have their own child cared for at their workplace, with no difference between the two professions (Figure 2C).

**Figure 3:**
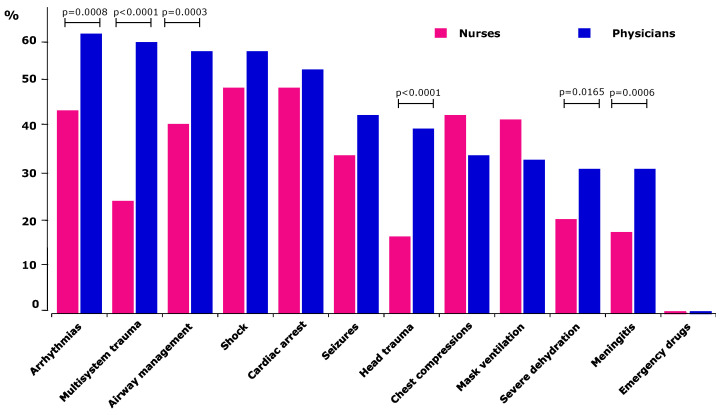
Perceived training needs in twelve specific areas and clinical scenarios. The bars depict the percentage (Oy axis) of nurses and physicians wishing to train the listed skills (Ox axis). When a significant difference was detected between nurses and physicians, the corresponding *p*-value is provided. The absence of any sign above the bars implies that no statistically significant difference between nurses and physicians was detected. Physicians, significantly more often than nurses, sought training for arrhythmias, airway management, multisystem trauma, head trauma, severe dehydration management and treatment of a child with acute meningitis. *Physicians* (*blue bars*): arrhythmias (*p* < 0.0004), multisystem trauma (*p* < 0.0014), airway management (*p* < 0.003), shock (*p* < 0.0043) and cardiac arrest (*p* < 0.03) were significantly more often cited than head trauma or the following depicted skills. *Nurses* (*red bars*): shock (*p* < 0.0001), cardiac arrest (*p* < 0.0001), arrhythmias (*p* < 0.0147), chest compressions (*p* < 0.0278) and mask ventilation (*p* < 0.0414) were more often cited than seizures, multisystem trauma, severe dehydration, acute meningitis, head trauma and emergency drugs.

When asked which skills and clinical scenarios respondents would like to train for (Figure 3), physicians most frequently mentioned the need to train their management skills of children with arrhythmias (62% of participant physicians), multisystem trauma (60%), shock (58%), cardiac arrest (54%) and airway management (58%). Nurses most frequently reported the need to practice caring for a child presenting with shock (50% of respondent nurses) or cardiac arrest (50%), arrhythmias (45%), requiring cardiopulmonary resuscitation (44%) or mask ventilation (43%). Physicians expressed their desire to train for arrhythmias, airway management, multisystem trauma, head trauma, management of severe dehydration and treatment of a child with acute meningitis significantly (*p* < 0.02) more often than nurses (Figure 3).

## 4. Discussion

This survey offers three main results. (1) Respondents perceive themselves well prepared to recognize a child’s clinical deterioration. (2) Both pediatric nurses and physicians rate their theoretical understanding of how to perform cardiopulmonary resuscitation higher than their actual practical preparedness, with cardiopulmonary resuscitation preparedness being higher among nurses than physicians. (3) Arrhythmias, shock, cardiac arrest and airway management constitute topics healthcare providers feel the need to train in the most, with physicians also citing multisystem trauma and nurses voicing chest compressions. 

This simple but straightforward cross-sectional survey study bares self-evident clinical relevance. These preliminary data on nurses’ and physicians’ skills and needs assessment offer guidance on what to emphasize in continuing training programs. The potential impact of tailored interventions on improving acute medical care and subsequent patient outcome and survival is considerable [3]. The identified gap between theoretical understanding and instant practical preparedness is not surprising and has, for example, recently also been described among Dutch and Belgian pediatricians [16]. A priority in medical training should therefore be to seek opportunities for narrowing this gap. Among other training approaches, medical simulation might offer a proven track to effectively tackle this gap [2,3]. This has, for example, been reported in the context of the current SARS-CoV-2 pandemic, where 80% of participants described improvements in clinical preparedness after simulation scenarios [17]. Finally, our survey also offers preliminary data to develop hypotheses to be tested in future research. 

Certain results deserve a closer look.

(1) At first glance, the gap in training perception between nurses and physicians might be difficult to explain. However, importantly, the work experience of respondent nurses was significantly longer than that of participating physicians (Table 1). Furthermore, in Switzerland, nurses enjoy patient exposure at an earlier training stage compared to physicians, and the nurse–patient ratio is higher than the physician-patient ratio.
(2) Second, the fact that almost all participants felt safe to have their own child cared for at the institution they work for is important and suggests a high level of institutional confidence. This in itself has been reported to be a patient safety promoter [18,19].
(3) Finally, the need for training appeared to favor certain clinical scenarios. On one side, it is remarkable that three out of the four most commonly cited areas of need (arrhythmias, cardiac arrest and shock) pertain to the domain of pediatric cardiology and intensive care. This might relate to the fact that these are rare but potentially life-threatening conditions. On the other side, emergency drugs were never cited as a training need. This is surprising, considering that medication errors are among the most potent contributors to iatrogenic morbidity and mortality [20]. An explanation might be that this is a global medical skill rather than a specific clinical scenario.

This cross-sectional study was able to take a snapshot of the self-perceived acute care readiness and training needs among pediatric nurses and physicians and presents some relevant strengths. First, it was conducted at a tertiary-level university children’s hospital, which provides care in all pediatric sub-specialties. Second, the respondent rate exceeded 50%, which is relatively high for a voluntary survey. Furthermore, the sample size surpassing 450 respondents is remarkable and greater than that of comparable studies performed in the past [21]. Third, both nurses and physicians were interviewed, allowing an interprofessional assessment. This study, however, also presents notable limitations. First, the survey investigated self-reported, subjectively perceived preparedness and skills and training needs in the absence of direct observation. Second, it is possible that participants might have been more interested in the topic than non-respondents, so selection bias cannot be excluded. Third, this monocentric study was performed at a single university hospital. Thus, the results cannot be extrapolated to other institutions and healthcare systems. Fourth, notwithstanding the globally respectable response rate, this figure was only moderate among physicians (<40%). Nevertheless, this is comparable to other voluntary surveys among physicians [13,15,22]. Finally, further studies are needed before training recommendations can be issued.

## 5. Conclusions

In conclusion, although nurses and physicians felt capable of recognizing a deteriorating child, both professional groups felt better prepared in theory than in practice. These results are important and suggest that future medical education and training should make it a priority to focus on narrowing this gap. This study might contribute to the design of future studies and the improvement of training in pediatric institutions.

## Figures and Tables

**Table 1:** Demographic and baseline characteristics of respondents. *p*-values refer to the comparison between nurses and physicians.

	All	Nurses	Physicians	*p*-Value
Department	469	315	154	*p* < 0.0001
Pediatrics	288 (61%)	218 (69%)	70 (45%)	
Pediatric surgery	82 (18%)	58 (18%)	24 (16%)	
Pediatric emergency	43 (9%)	28 (8.9%)	15 (9.7%)	
Other (e.g., across several departments)	56 (12%)	11 (3.5%)	45 (29%)	
Work experience as a healthcare professional	469	315	154	*p* = 0.0002
<1 year	3 (0.6%)	2 (0.6%)	1 (0.6%)	
1–5 years	99 (21%)	58 (18%)	41 (27%)	
6–10 years	95 (20%)	52 (17%)	43 (28%)	
>10 years	272 (58%)	203 (64%)	69 (45%)	
Duration of employment at current hospital	468	314	154	*p* < 0.0001
<1 year	57 (12%)	17 (5.4%)	40 (26%)	
1–5 years	152 (33%)	94 (30%)	58 (38%)	
6–10 years	72 (15%)	46 (15%)	26 (17%)	
>10 years	187 (40%)	157 (50%)	30 (19%)	
Hours spent working on a weekly basis	469	315	154	*p* < 0.0001
<20 h/week	100 (21%)	70 (22%)	30 (19%)	
20–40 h/week	145 (31%)	123 (39%)	22 (14%)	
>40 h/week	224 (48%)	122 (39%)	102 (66%)	
Exposure frequency to life-threatening situations	459	309	150	*p* = 0.0005
daily	81 (18%)	53 (17%)	28 (19%)	
weekly	79 (17%)	41 (13%)	38 (25%)	
monthly	87 (19%)	53 (17%)	34 (23%)	
once every 2–6 months	104 (23%)	77 (25%)	27 (18%)	
yearly	62 (14%)	46 (15%)	16 (11%)	
<1 time/year	46 (10%)	39 (13%)	7 (4.7%)	

## Data Availability

The data presented in this study are available from the corresponding author upon reasonable request.

## Supplementary Materials

The following supporting information can be downloaded at: https://www.mdpi.com/article/10.3390/children9020271/s1, Table S1: Survey instrument.
