# Supplementary material for: Preparedness for Life-Threatening Situations in a Pediatric Tertiary-Care University Children’s Hospital: A Survey
Source: Children (Basel). 2022 Feb 16;9(2):271. doi: 10.3390/children9020271 (PMC8870381; doi:10.3390/children9020271)
Supplement: Supplementary file 1 [file children-09-00271-s001.zip › children-1558357-supplementary.pdf]

Supplementary material

Survey instrument

Q1 How many years of professional experience does your career cover ?
a) <1y
b) 1-5y
c) 6-10y
d) >10y

Q2 How long have you been working at the University Children's Hospital of Berne?
a) <1y
b) 1-5y
c) 6-10y
d) >10y

Q3 On average, how many hours a week do you work at the University Children's Hospital of Berne?
a) <20 h/W
b) 20-39 h/W
c) >40 h/W

Q4 Approximately, how often do you encounter life threatening situations in your clinical routine ?
a) Daily
b) Weekly
c) About once a month
d) Every 2 to 6 months
e) About once a year
f) Less than once a year or never

Q5 How do you rate your ability to recognize a child's deteriorating condition ?
a) very good
b) good
c) satisfactory
d) poor
e) very poor

Q6 In theory, how well prepared do you feel to partake in an emergency requiring resuscitation?
a) Very well
b) Well
c) Satisfactory
d) Poor
e) Very poor

Q7 How well prepared do you feel to actively partake in an emergency requiring resuscitation?

a) Very well
b) Well
c) Satisfactory
d) Poor
e) Very poor

Q8 I feel comfortable operating medical devices used in my work environment.

a) strongly agree
b) agree
c) unsure/I do not know
d) disagree
e) strongly disagree

Q9 I would feel comfortable having my child treated where I work.

a) strongly agree
b) agree
c) neutral
d) disagree
e) strongly disagree

Q10 Which of the following medical emergencies and skills would you like to receive specific training? (multiple replies are possible)

a) Arrhythmias
b) Multisystem trauma
c) Airway management
d) Shock
e) Cardiac arrest
f) Seizures
g) Head trauma
h) Chest compressions
i) Bag mask ventilation
j) Severe dehydration
k) Meningitis
l) Prescribing and/or administering emergency drugs
